# Why 'down under' is a cut above: a comparison of rates of and reasons for caesarean section in England and Australia

**DOI:** 10.1186/1471-2393-14-149

**Published:** 2014-04-26

**Authors:** Samantha J Prosser, Yvette D Miller, Rachel Thompson, Maggie Redshaw

**Affiliations:** 1 School of Psychology, The University of Queensland, Brisbane, Australia; 2 School of Public Health & Social Work, Queensland University of Technology, Brisbane, Australia; 3 The Dartmouth Center for Health Care Delivery Science, Dartmouth College, Hanover, NH, USA; 4 Policy Research Unit for Maternal Health and Care, National Perinatal Epidemiology Unit, University of Oxford, Oxford, UK

**Keywords:** Caesarean section, Childbirth, Pregnancy, Cross-cultural comparison, Vaginal birth after caesarean, Previous caesarean section, Patient-reported data, Quality improvement

## Abstract

**Background:**

Most studies examining determinants of rising rates of caesarean section have examined patterns in documented reasons for caesarean over time in a single location. Further insights could be gleaned from cross-cultural research that examines practice patterns in locations with disparate rates of caesarean section at a single time point.

**Methods:**

We compared both rates of and main reason for pre-labour and intrapartum caesarean between England and Queensland, Australia, using data from retrospective cross-sectional surveys of women who had recently given birth in England (*n* = 5,250) and Queensland (*n* = 3,467).

**Results:**

Women in Queensland were more likely to have had a caesarean birth (36.2%) than women in England (25.1% of births; *OR* = 1.44, *95*% *CI* = 1.28-1.61), after adjustment for obstetric characteristics. Between-country differences were found for rates of pre-labour caesarean (21.2% vs. 12.2%) but not for intrapartum caesarean or assisted vaginal birth. Compared to women in England, women in Queensland with a history of caesarean were more likely to have had a pre-labour caesarean and more likely to have had an intrapartum caesarean, due only to a previous caesarean. Among women with no previous caesarean, Queensland women were more likely than women in England to have had a caesarean due to suspected disproportion and failure to progress in labour.

**Conclusions:**

The higher rates of caesarean birth in Queensland are largely attributable to higher rates of caesarean for women with a previous caesarean, and for the main reason of having had a previous caesarean. Variation between countries may be accounted for by the absence of a single, comprehensive clinical guideline for caesarean section in Queensland.

## Background

Since the early 1990s, the proportion of women giving birth by caesarean section has risen steadily, with an average relative increase of 48.9% between 1992 and 2007 across 18 developed countries [1]. While for almost three decades the World Health Organization has recommended that the rate of caesarean section need not exceed 10-15% of all births [2], it has recently acknowledged the absence of empirical evidence to support an optimal rate of caesarean [3]. In 2007 the rate of caesarean section was greater than 30% of all births in countries including Australia, Italy, Portugal, Switzerland, and the United States [1]. Caesarean section may be a life-saving procedure, but it also carries risks. Compared with vaginal birth, caesarean section may place women and their babies at increased odds of morbidity or mortality at the time of birth [4,5], may have negative implications for future pregnancies and maternal health [6-8], and requires additional maternity care resources [9,10]. The extent to which there is a net benefit of caesarean section depends on how often, when, and why the procedure is used. Thus, understanding the factors driving the increasing caesarean rate is an important part of efforts to prevent potential over-use of caesarean section and ensure a net benefit for women, babies, and society.

Currently consensus is lacking on the most significant contributors to the increasing caesarean rate. Proposed determinants fall into three broad domains which are not mutually exclusive. Firstly, some arguments focus on changes in maternal and obstetric characteristics (e.g., older maternal age, greater pre-pregnancy weight, higher prevalence of nulliparity) that have increased the risk of perinatal complications [11]. However, several studies have suggested that maternal and obstetric risk factors alone do not account for the increasing caesarean rate [12-14]. Other arguments focus on maternal request for caesarean section in the absence of a medical indication as a factor driving the increasing caesarean rate [15]. Despite its popularity, empirical support for this explanation is limited [16,17]; research has found that maternal request caesareans represent only 8% of the *increase* in rates of primary caesarean section [18] and only 4% of *all* primary caesareans [19]. Finally, some arguments focus on changes in obstetric practice in explaining the increasing caesarean rate. For example, breech presentation, multiple pregnancy, and previous caesarean have become widely recognised as indications for caesarean [20,21]. However, the rate of increase of the prevalence of these factors in the birthing population is not sufficient to fully account for the increasing caesarean rate [22]. Moreover, the increasing rate of primary caesareans and decreasing attempts at vaginal birth after caesarean (VBAC) contribute to higher rates of repeat caesarean [17,23].

To date, most studies examining determinants of the increasing caesarean rate have examined patterns in documented reasons for caesarean over time in a single location. An alternative approach is to conduct cross-cultural research that examines practice patterns in locations with disparate rates of caesarean section at a single time point. In particular, potentially informative comparisons can be made between Australia and England. These two countries have a number of socio-economic similarities; both are affluent countries, have well-established publically-funded health care systems, and have guiding bodies tasked with ensuring quality and safety in health care. Despite their similarities, there are differences between Australia and the UK in the caesarean rate. In the UK, the caesarean rate for 2009–10 was 24.8% [24], while in Australia for 2010 it was 31.5% [25].

A possible explanation for these disparate caesarean rates is differences between Australia and the UK in how labour and birth care is managed. These differences are not overt, as available clinical guidelines relevant for decision-making about caesarean section are largely congruent between the countries [21,26-29]. Rather, it may be that differences in rates of caesarean between Australia and the UK reflect the extent to which contraindications for vaginal birth are agreed upon and applied in uncertain situations. If so, it is likely that differences would also be apparent in the frequency with which specific reasons for caesarean section are recorded. Comparison of reasons for both types of caesarean section – intrapartum and pre-labour – and among specific sub-populations of women (e.g. women with a previous caesarean), is likely to provide insight into how care decisions affect the rates of caesarean in Australia and the UK.

The aim of this study was to examine differences in the rates of pre-labour and intrapartum caesarean for women in England and Queensland, Australia, and to determine whether there are differences in reported reasons for caesarean birth, using retrospective, self-reported data. The congruence of maternal self-report with clinical report or hospital records for perinatal events has been demonstrated exhaustively, particularly for mode of birth and caesarean section [30-32]. Additionally, maternal and clinical reports of reasons for caesarean have a concordance rate of around 90% [33]. Specifically, we sought to compare the single (main) reason for caesarean across countries, separated by timing of caesarean (intrapartum or pre-labour) and type of caesarean (primary or repeat), to identify possible contributing factors to the discrepant overall rates.

## Method

### Background

Researchers in both the National Perinatal Epidemiology Unit in Oxford, England, and the Queensland Centre for Mothers & Babies in Queensland, Australia, conduct population surveys of women’s experiences of maternity care. The surveys provide an opportunity to assess the state of maternity care services from a consumer perspective at a given point in time and to examine changes over time in service delivery. Although some specific survey items differ, both instruments retrospectively assess women’s experiences of care during pregnancy, labour, birth and after birth, in addition to obstetric history and demographic characteristics. Ethical approval for these surveys and subsequent analyses was provided respectively by the *Trent Multicentre Research Ethics Committee* in England and The University of Queensland *Behavioural & Social Sciences Ethical Review Committee*.

### Participants and procedures

#### England

A random sample of 10,000 women, aged at least 16 years, who had a live birth in England over a two-week period in October-November 2009, were invited to complete the survey. The Office for National Statistics drew the sample based on birth registration records and was responsible for mailing out the surveys. Each woman was initially sent a survey when her baby was approximately three months old. Using a tailored reminder system, women who had not completed the survey were sent a reminder two weeks later, an additional questionnaire four weeks later, and a final reminder letter four weeks after that. Surveys could be returned by mail or completed online. Further detail on the sampling methodology is provided elsewhere [34].

#### Queensland

All women who had a live birth in Queensland (a state of Australia) in a two-month period (February to March 2010), and who were not found to have had a baby that died since birth, were invited to complete the extended (24-page) *Having a Baby in Queensland Survey,* 2010. The sample for this survey was drawn from databases of compulsory birth notification and registration records held by the *Queensland Registry of Births, Deaths and Marriages (BDM)*. The entire eligible population was sent a survey package four to five months after birth by *BDM*. Women could (i) complete and return the paper survey using a reply-paid envelope, (ii) complete the same survey online, or (iii) complete an abbreviated survey via telephone with a female interviewer and, if necessary, a translator. All women were sent a reminder to complete the survey two weeks after the initial mailing. Again, further detail on the sampling methodology is provided elsewhere [35,36].

### Measures

#### Mode of birth

The survey in England asked ‘*Thinking about the birth of your baby this time, what kind of delivery did you have?*’ with four response options: normal (vaginal) birth, a caesarean (through a cut in the abdomen), delivery using forceps, and delivery using vacuum cap on the baby’s head (ventouse). The Queensland survey asked ‘*How was your baby born?*’ with five response options: an unassisted vaginal birth, a vaginal birth assisted with forceps, a vaginal birth assisted with a vacuum, a vaginal birth assisted by forceps and a vacuum, and a caesarean birth. For the current study, a four category mode of birth variable was created whereby vaginal birth was further categorised as assisted (forceps and/or vacuum) or unassisted, and caesarean birth was further categorised as intrapartum (performed after the onset of labour) or pre-labour (performed before the onset of labour). To determine whether women experienced labour, women in England were asked ‘*Did you have a labour?*’ and indicated ‘*yes*’ or ‘*no*’. The Queensland survey asked, ‘*Did you or someone else try to induce your labour?*’ with four response options: No, my labour started by itself; Yes, and it worked; Yes, but it didn’t work; No, I didn’t have a labour. Responses to the first two options were coded as having experienced labour and the latter two options were indicative of not having experienced labour.

#### Reasons for caesarean

Both surveys asked women to indicate why they had a caesarean from a checklist of possible reasons (see Table 1). The checklists were similar between the surveys; however, only the Queensland checklist included the reasons of ‘carer recommendation’ and ‘hospital policy’ and only the England checklist included the reason of ‘multiple pregnancy’. Women could specify multiple reasons.

**Table 1:** Checklists of possible reasons for caesarean

**Reason**	**England survey items**	**Queensland survey items**
	***Why did you have a caesarean?***	***Why did you have a caesarean birth?***
Previous caesarean	*Because I had a caesarean before*	*I have had a caesarean birth before*
Maternal preference	*I wanted my baby to be born this way*	*I wanted my baby to be born this way*
Fetal distress	*My baby was ‘distressed’*	*My baby was ‘distressed’*
Failure to progress in labour	*Labour had ‘failed to progress’*	*My labour had ‘failed to progress’*
Recommendation	*-*	*It was recommended by my care provider*
Suspected disproportion	*My baby wouldn’t fit though my pelvis*	*My baby wouldn’t fit though my pelvis*
Breech presentation	*Breech presentation (feet first)*	*My baby was breech (feet or bottom first)*
Maternal health concerns	*Because of worries about my health*	*Because of worries about my health (e.g. placenta praevia or pre-eclampsia)*
Premature labour	*Because I was in premature labour*	*I was in premature labour*
Multiple pregnancy	*I had twins or triplets*	*-*
Recommendation	*-*	*It was hospital policy*
	*Other reason_________*	*Other:__________*
	*Don’t know/Can’t remember*	*Don’t know*

Where possible, open-text ‘other’ responses were back-coded into one of the specified reasons for caesarean. Based on these ‘other’ responses, we created the ‘carer recommendation’ and ‘hospital policy’ categories for women from England and the ‘multiple pregnancy’ category for women from Queensland. Three additional unique categories were created: *obstetric history* (other than previous caesarean), *fetal health concerns* (other than fetal distress which was limited to heart rate concerns), and *malpresentation* (other than breech presentation). Open-text reasons occurring at a low frequency remained coded as *other*.

To allow comparison between the two countries, a variable representing a single (main) reason for the caesarean was created, according to a hierarchical algorithm developed by the authors [see Additional file 1]. Reasons of ‘hospital policy’ or ‘carer recommendation’ were combined as *recommendation.* Women with a history of caesarean section were only classified with *previous caesarean* as the reason for their most recent caesarean if this was the sole reason selected, or if this reason was selected along with *maternal preference* or *recommendation*. Where women had selected *maternal preference* and *recommendation* as the only reasons for their caesarean, the single reason for caesarean was coded as *shared preference*. For women who experienced labour prior to their caesarean, ‘failure to progress’, ‘suspected disproportion’ and ‘malpresentation’ were grouped as *failure to progress in labour*.

#### Previous caesarean

For both countries, we used other survey responses to create a dichotomous variable representing whether or not women had previously given birth by caesarean. We also created a three-category version that accounted for parity (*primiparous, multiparous without a previous caesarean, multiparous with a previous caesarean*) for the purpose of comparing across countries.

#### Other measures

Maternal age, maternal country of birth, plurality, gestational age, infant birthweight, and obstetric risk factors (gestational diabetes (GDM), hypertension or pre-eclampsia, placental complications, and other risk factors) were assessed comparably in both surveys. For maternal education, we coded women in England as not having completed secondary education if they were 16 years or less when they left full-time education, and women in Queensland, if they had no formal qualifications or their highest level of education was year 10 or equivalent.

### Analytic strategy

Inclusion in the current study required women to have complete data for mode of birth and previous caesarean. Using chi-square analyses, we compared maternal, obstetric and infant characteristics between England and Queensland. Obstetric characteristics that differed significantly by country were entered as covariates in logistic regression analyses to compare mode of birth between the countries. All variables were entered simultaneously and comparisons were conducted on the overall samples, and then separately for women with and without a previous caesarean birth.

For both countries, the proportion of women that experienced a caesarean birth for each specific reason was calculated as a proportion of all women with caesarean births and as a proportion of all women who gave birth (i.e., irrespective of mode of birth). To determine whether the absolute rates for each specific reason differed by country, comparisons of reasons for caesarean were conducted relative to all women who gave birth. Logistic regression analyses were conducted separately for primary caesarean (i.e., women having their first caesarean) and repeat caesarean, and by timing of caesarean (pre-labour or intrapartum), and included obstetric characteristics that differed significantly by country as covariates. For some specific comparisons, the small number of cases for certain obstetric characteristics (e.g., GDM, placental complications, high blood pressure/pre-eclampsia, and plurality) prevented adjustment for those variables. Given the lack of variance in those indicators for relevant analyses, their omission is unlikely to substantively influence the findings. Alpha was set at 0.05 for all statistical comparisons.

## Results

### Survey respondents

In England, 9,851 women were assumed to have received the survey. Overall, 5,332 completed surveys were returned, resulting in a 54.1% usable response rate. Relative to the 2009–2010 birthing population in England, survey respondents were approximately representative of women in terms of maternal country of birth, plurality, total number of births, area of residence, having experienced labour, and mode of birth (11.5% of births were by caesarean without labour and 12.3% were by caesarean with labour) [24]. Women aged 35 years or older were over-represented in the sample, while women belonging to certain ethnic groups (Asian or Asian British, Black or Black British, or Chinese and other) were under-represented. Comparison data were unavailable for previous caesarean.

In Queensland, 10,346 eligible women were assumed to have received the survey. Overall, 3,542 completed surveys were returned (2,990 mail, 540 online, 12 telephone), resulting in a 34.2% usable response rate. Survey respondents were approximately representative of the Queensland birthing population in 2010 for maternal country of birth, the proportion aged 35 years or older, parity, experiencing labour, mode of birth (20.7% of births were by caesarean without labour and 12.2% were by caesarean with labour), and previous caesarean (17.9% of women) [25]. The sample under-represented birthing women who identify as Aboriginal and/or Torres Strait Islander and over-represented women who gave birth in a private hospital or who had a multiple pregnancy. Additional details are reported elsewhere [37].

### Sample characteristics

Complete data was available for 5,250 women in England and 3,467 women in Queensland. Demographic characteristics by country are provided in Table 2. No differences were found between the countries in the proportions of women aged 35 years or older, the proportions born in the country of their index birth experience, or in infant birthweight. Women in Queensland were more likely than women in England to have completed secondary education, to have previously given birth by caesarean, to have had a multiple pregnancy, to have given birth prior to 37 weeks’ gestation, and to have reported gestational diabetes, placental complications, hypertension/pre-eclampsia, and other pregnancy risk factors.

**Table 2:** Maternal, obstetric and infant characteristics by country

	**England (N = 5,250)**		**Queensland (N = 3,467)**		***p***
	**N**	**%**	**N**	**%**	
Maternal Characteristics					
**Maternal Age**					.879
*34 years or younger*	3,863	74.3	2,568	74.4	
*35 years or older*	1,337	25.7	882	25.6	
*Missing*	*50*		*17*		
**Secondary Education**					< .001
*Completed*	3,972	76.8	3,107	90.3	
*Not completed*	1,201	23.2	334	9.7	
*Missing*	*77*		*26*		
**Maternal Country of Birth**					.444
*Same as index birth*	3,965	79.0	2,755	79.7	
*Different from index birth*	1,052	21.0	701	20.3	
*Missing*	*233*		*11*		
Obstetric Characteristics					
**Parity**					< .001
*Primiparous*	2,564	49.8	1,577	45.5	
*Multiparous – No previous CS*	1,999	38.8	1,266	36.5	
*Multiparous – Previous CS*	590	11.4	623	18.0	
*Missing*	*97*		*1*		
**Plurality**					< .001
*Single*	5,157	98.4	3,345	96.5	
*Multiple*	86	1.6	122	3.5	
*Missing*	*7*		*-*		
**Gestational Diabetes**					< .001
*Yes*	129	2.5	252	7.3	
*No*	5,101	97.5	3,205	92.7	
*Missing*	-		-		
**Placental Complications**					.010
*Yes*	309	5.9	252	7.3	
*No*	4,921	94.1	3,205	92.7	
*Missing*	-		-		
**Hypertension/Pre-eclampsia**					.012
*Yes*	402	7.7	318	9.2	
*No*	4,828	92.3	3,139	90.8	
*Missing*	*-*		*-*		
**Other Risk Factors**^**§**^					< .001
*Yes*	931	17.8	872	25.3	
*No*	4,299	82.2	2,571	74.7	
*Missing*	-		-		
Infant Characteristics					
**Gestational Age**					< .001
*Less than 37 weeks*	334	6.4	291	8.6	
*37 weeks or more*	4,852	93.6	3,096	91.4	
*Missing*	*64*		*80*		
**Birthweight**					.189
*Less than 2500 g*	273	5.4	195	5.8	
*2500 – 3999 g*	4,105	81.5	2,680	80.0	
*4000 g or more*	656	13.0	477	14.2	
*Missing*	*216*		*115*		

Note. ^**§**^Risk factors not already accounted for, such as fetal presentation, pre-labour rupture of membranes, threatened pre-term labour, intrauterine growth restriction, suspected fetal macrosomia, oligohydramnios or polyhydramnios, and complications in previous pregnancies or births.

### Mode of birth comparisons

Women in Queensland were more likely to have had a caesarean birth (36.2% of births) than women in England (25.1% of births; see Table 3). The rates of intrapartum caesarean and assisted vaginal birth, respectively, were similar across countries, but the rate of pre-labour caesarean was higher in Queensland than in England.

**Table 3:** Mode of birth by country

**Mode of birth**	**England (N = 5,250)**		**Queensland (N = 3,467)**		**OR**^**~**^	**95% CI**
	**N**	**%**	**N**	**%**		
All Women						
**Vaginal Birth**	**3,930**	**74.9**	**2,213**	**63.8**		
*Unassisted*	*3,269*	*62.3*	*1,830*	*52.8*		
*Assisted*	*661*	*12.6*	*383*	*11.0*	*0.94*	*0.82-1.08*
**Caesarean Birth**	**1,320**	**25.1**	**1,254**	**36.2**	1.44^***^	**1.28-1.61**
*Intrapartum*	*677*	*12.9*	*524*	*15.1*	*1.14*	*1.00-1.30*
*Pre-labour onset*	*643*	*12.2*	*730*	*21.1*	1.51^***^	*1.31-1.73*
Women without a previous Caesarean						
**Vaginal Birth**	**3,763**	**80.8**	**2,128**	**74.8**		
*Unassisted*	*3,140*	*67.4*	*1,766*	*62.1*		
*Assisted*	*623*	*13.4*	*362*	*12.7*	*0.98*	*0.85-1.13*
**Caesarean Birth**	**897**	**19.2**	**716**	**25.2**	1.33^***^	**1.18-1.50**
*Intrapartum*	*560*	*12.0*	*410*	*14.4*	1.21^*^	*1.05-1.40*
*Pre-labour onset*	*337*	*7.2*	*306*	*10.8*	1.36^***^	*1.14-1.62*
Women with a previous Caesarean						
**Vaginal Birth**	**167**	**28.3**	**85**	**13.7**		
*Unassisted*	*129*	*21.9*	*64*	*10.3*		
*Assisted*	*38*	*6.4*	*21*	*3.4*	0.54^*†^	*0.31-0.94*
**Caesarean Birth**	**423**	**71.7**	**536**	**86.4**	2.28^***^	**1.69-3.06**
*Intrapartum*	*117*	*19.8*	*114*	*18.3*	*0.90*	*0.67-1.20*
*Pre-labour onset*	*306*	*51.9*	*424*	*68.1*	1.87^***^	*1.47-2.37*

Note. ^*^p < .05, ^**^p < .01, ^***^p < .001. ^~^ The odds for women in Queensland (relative to women in England) of having had the specified mode of birth, adjusted for parity, plurality, GDM, placental complications, hypertension/pre-eclampsia, and other risk factors. ^†^ The odds of assisted delivery did not differ between England and Queensland when examined only among vaginal births (*OR* = 1.10, 95% *CI* = 0.59-2.06).

Among women without a previous caesarean, those in Queensland had higher odds of having had a caesarean section than those in England; this was consistent for both pre-labour and intrapartum caesareans (see Table 3).

Among women with a previous caesarean, those in Queensland had higher odds of having had a caesarean section than those in England. Women in Queensland had higher odds of having had a pre-labour caesarean, but not of intrapartum caesarean (see Table 3).

### Reasons for caesarean

Analyses relating to reasons for caesarean were adjusted for obstetric characteristics (i.e., parity, plurality, GDM, placental complications, hypertension/pre-eclampsia, and other risk factors). Women with missing data (England, *N* = 8; Queensland, *N* = 3) or indicating ‘*don’t know*’ (England, *N* = 0; Queensland, *N* = 1) for reasons for caesarean were excluded, leaving usable data for 1,312 women from England and 1,250 women from Queensland.

On average, women provided 1.76 (*SD* = 0.87; range = 1–7) reasons for their caesarean. Women in Queensland provided more reasons (*M* = 1.91, *SD* = 0.94) than women in England (*M* = 1.62, *SD* = 0.77; *t* (2408.93) = 8.51, *p* < .001). The majority of women (74.4% from Queensland and 87.0% from England) provided 1 or 2 reasons for their caesarean. Overall, 21.1% of women in Queensland and 30.6% of women in England provided open-text comments for ‘other reasons’.

### Reasons for pre-labour caesarean

The most common reasons for pre-labour caesarean were breech presentation or maternal heath concerns in England, and previous caesarean in Queensland (see Table 4). Women in Queensland were more likely than women in England to have had a pre-labour caesarean due to a previous caesarean, suspected disproportion, or maternal preference, but were less likely to have had a pre-labour caesarean due to fetal distress or multiple pregnancy.

**Table 4:** Single (main) reason for pre-labour caesarean, by previous caesarean and country

**Reasons for caesarean**	**All women**					**No previous caesarean**					**Previous caesarean**				
	**England**		**Queensland**		**OR (95% CI)**^**+**^	**England**		**Queensland**		**OR (95% CI)**^**+**^	**England**		**Queensland**		**OR (95% CI)**^**+**^
	**% of PLCS (N = 642)**	**% of all births** ** (N = 5,242)**	**% of PLCS (N = 726)**	**% of all births (N = 3,463)**		**% of PLCS (N = 336)**	**% of all births**^**^ **^**(N = 4,655)**	**% of PLCS (N = 305)**	**% of all births**^**^ **^**(N = 2,843)**		**% of PLCS (N = 306)**	**% of all births**^**# **^**(N = 587)**	**% of PLCS (N = 421)**	**% of all births**^**# **^**(N = 620)**	
*Previous Caesarean Only*	17.1	2.1	30.9	6.5	3.40 (2.69-4.30)						35.9	18.7	53.2	36.1	2.82 (2.15-3.70)
*Breech Presentation*	24.0	2.9	16.0	3.4	0.95 (0.73-1.23)	37.2	2.7	29.5	3.2	1.02 (0.76-1.36)	9.5	4.9	6.2	4.2	0.73 (0.42-1.28)
*Maternal Health Concerns*	23.4	2.9	18.6	3.9	0.90 (0.69-1.17)	22.9	1.7	24.9	2.7	1.40 (1.00-1.97)	23.9	12.4	14.0	9.5	0.49 (0.33-0.74)
*Suspected Disproportion*	8.6	1.0	10.6	2.2	1.60 (1.12-2.30)	5.1	0.4	10.8	1.2	2.78 (1.53-5.05)	12.4	6.5	10.5	7.1	1.16 (0.74-1.83)
*Fetal Distress*	8.4	1.0	1.2	0.3	0.22 (0.11-0.44)	12.2	0.9	2.3	0.3	0.27 (0.12-0.60)	4.2	2.2	0.5	0.3	0.10 (0.02-0.46)
*Carer/Hospital Recommendation*	-	-	6.5	1.4	-	-	-	10.8	1.2	-	-	-	3.3	2.3	-
*Maternal Preference*	3.4	0.4	4.1	0.9	2.03 (1.16-3.56)	3.0	0.2	8.5	0.9	4.47 (2.14-9.33)	3.9	2.0	1.0	0.7	0.33 (0.11-1.04)
*Obstetric History*	4.4	0.5	3.7	0.8	1.08 (0.63-1.87)	3.9	0.3	4.3	0.5	1.58 (0.73-3.44)	4.9	2.6	3.3	2.3	0.75 (0.35-1.61)
*Fetal Health Concerns*	1.9	0.2	2.2	0.5	1.37 (0.63-2.97)	2.4	0.2	3.0	0.3	1.44 (0.54-3.83)	1.3	0.7	1.7	1.1	1.09 (0.30-3.94)
*Malpresentation*	0.9	0.1	0.7	0.1	0.97 (0.28-3.34)	1.2	0.1	1.3	0.1	1.08 (0.25-4.64)	0.7	0.3	0.2	0.2	0.40 (0.04-4.50)
*Premature Labour*	0.8	0.1	0.4	0.1	0.52 (0.12-2.29)	0.9	0.1	-	-	-	0.7	0.3	0.7	0.5	1.02 (0.16-6.50)
*Shared Preference*	-	-	2.6	0.6	-	-	-	1.0	0.1	-	-	-	3.8	2.6	-
*Multiple Pregnancy*	2.2	0.3	0.1	0.0	0.09 (0.01-0.72)	3.3	0.2	0.3	0.0	0.14 (0.02-1.10)	1.0	0.5	-	-	-
*Other*	5.0	0.6	2.3	0.5	0.80 (0.43-1.47)	8.8	0.6	3.3	0.4	0.65 (0.30-1.37)	1.6	0.9	1.7	1.1	1.32 (0.41-4.25)

Note. PLCS = Pre-labour caesarean section. ^+^ The odds for women in Queensland (relative to women in England) of having had a caesarean for the specified reason. ^^^ The total number of women without a previous caesarean (irrespective of mode of birth for index pregnancy). ^#^ The total number of women with a previous caesarean (irrespective of mode of birth).

#### Pre-labour caesarean for women with no previous caesarean

In both countries, breech presentation and maternal health concerns were the most common reasons for pre-labour caesarean among women without a previous caesarean. While not provided as a response option for women in England (and not spontaneously reported by women in their ‘other’ responses as the primary reason), 10.8% of Queensland women with no previous caesarean births had a pre-labour caesarean solely because it was recommended by their care provider or hospital. Women in Queensland were less likely than women in England to have had a pre-labour caesarean due to fetal distress but were more likely to have had a pre-labour caesarean due to suspected disproportion or maternal preference.

#### Pre-labour caesarean for women with a previous caesarean

Among women with a previous caesarean, the most common reasons for pre-labour caesarean in England and Queensland were previous caesarean and maternal health concerns. Women in Queensland were more likely than women in England to have had a pre-labour caesarean due to previous caesarean but less likely to have had a pre-labour caesarean due to maternal health concerns or fetal distress.

### Reasons for intrapartum caesarean

The most common reasons for intrapartum caesarean in England and Queensland were failure to progress in labour and fetal distress (see Table 5). Women in Queensland were more likely than women in England to have had an intrapartum caesarean due to failure to progress in labour, premature labour or due to previous caesarean, but were less likely to have had an intrapartum caesarean due to fetal distress.

**Table 5:** Single (main) reason for intrapartum caesarean, by previous caesarean and country

**Reasons for caesarean**	**All women**					**No previous caesarean**					**Previous caesarean**				
	**England**		**Queensland**		**OR (95% CI)**^**+**^	**England**		**Queensland**		**OR (95% CI)**^**+**^	**England**		**Queensland**		**OR (95% CI)**^**+**^
	**% of ICS (N = 670)**	**% of all births (N = 5,242)**	**% of ICS (N = 524)**	**% of all births (N = 3,463)**		**% of ICS (N = 556)**	**% of all births**^**^ **^**(N = 4,655)**	**% of ICS (N = 410)**	**% of all births**^**^ **^**(N = 2,843)**		**% of ICS (N = 114)**	**% of all births**^**# **^**(N = 587)**	**% of ICS (N = 114)**	**% of all births**^**# **^**(N = 620)**	
*Previous Caesarean Only*	1.9	0.3	6.7	1.0	4.17 (2.19-7.94)						11.4	2.2	30.7	5.7	2.76 (1.44-5.30)
*Fetal Distress*	48.5	6.2	32.4	4.9	0.77 (0.63-0.94)	51.6	6.2	38.0	5.5	0.86 (0.70-1.06)	33.3	6.5	12.3	2.3	0.31 (0.17-0.59)
*Failure to Progress in Labour*	33.9	4.3	41.6	6.3	1.50 (1.23-1.83)	33.6	4.0	43.2	6.2	1.64 (1.32-2.04)	35.1	6.8	36.0	6.6	1.00 (0.64-1.59)
*Breech Presentation*	10.7	1.4	9.2	1.4	0.87 (0.59-1.28)	11.3	1.4	9.8	1.4	0.91 (0.60-1.38)	7.9	1.5	7.0	1.3	0.69 (0.25-1.89)
*Maternal Health Concerns*	2.1	0.3	3.4	0.5	1.49 (0.73-3.05)	0.9	0.1	2.9	0.4	3.43 (1.19-9.84)	7.9	1.5	5.3	1.0	0.63 (0.22-1.81)
*Shared Preference*	-	-	0.4	0.1	-	-	-	0.2	0.0	-	-	-	0.9	0.2	-
*Carer/Hospital Recommendation*	-	-	1.5	0.2	-	-	-	1.7	0.3	-	-	-	0.9	0.2	-
*Obstetric History*	0.4	0.1	0.2	0.0	0.50 (0.05-4.85)	0.5	0.1	-	-	-	-	-	0.9	0.2	-
*Premature Labour*	0.3	0.0	2.5	0.4	11.80 (1.51-92.30)	0.2	0.0	1.5	0.2	5.81 (0.67-50.48)	-	-	6.1	1.1	-
*Fetal Health Concerns*	0.4	0.1	1.1	0.2	2.49 (0.61-10.11)	0.5	0.1	1.5	0.2	2.68 (0.66-10.88)	-	-	-	-	-
*Maternal Preference*	0.3	0.0	0.4	0.1	0.80 (0.11-6.05)	-	-	0.5	0.1	-	1.8	0.3	-	-	-
*Multiple Pregnancy*	0.9	0.1	0.2	0.0	0.21 (0.03-1.81)	0.7	0.1	0.2	0.0	0.37 (0.04-3.32)	1.8	0.3	-	-	-
*Other*	0.6	0.1	0.4	0.1	0.77 (0.14-4.26)	0.5	0.1	0.5	0.1	1.05 (0.18-6.31)	0.9	0.2	-	-	-

Note. ICS = Intrapartum caesarean section. ^+^The odds for women in Queensland (relative to women in England) of having had a caesarean for the specified reason. ^^^The total number of women without a previous caesarean (irrespective of mode of birth for index pregnancy). ^#^The total number of women with a previous caesarean (irrespective of mode of birth).

#### Intrapartum caesarean for women with no previous caesarean

Fetal distress and failure to progress in labour were the most common reasons for intrapartum caesarean in England and Queensland, among women without a previous caesarean. Women in Queensland were more likely than women in England to have had an intrapartum caesarean due failure to progress in labour or due to maternal health concerns.

#### Intrapartum caesarean for women with a previous caesarean

Among women with a previous caesarean, the most common reason for intrapartum caesarean in England and Queensland was failure to progress in labour. Women in Queensland were more likely the women in England to have had an intrapartum caesarean due to previous caesarean and less likely to have had an intrapartum caesarean due to fetal distress.

## Discussion

This study sought to compare self-reported rates of, and reasons for, caesarean section between England and Queensland (Australia) to identify potential explanations for discrepant caesarean section rates. Overall, women in Queensland were at higher odds of having a caesarean section than women in England; they had approximately 1.5 times higher odds of a pre-labour caesarean, and those with a history of caesarean had more than double the odds of having had a caesarean. While the overall rate of intrapartum caesareans did not differ between the countries, rates were higher in Queensland than in England among women without a previous caesarean. The absence of any difference between countries in the rate of assisted vaginal deliveries suggests that England’s lower caesarean rate is not explained by a tendency to choose assisted vaginal delivery over emergency intrapartum caesarean.

In both countries, the most commonly reported reasons for pre-labour caesarean were breech presentation for women without a previous caesarean and previous caesarean for women with a history of caesarean. Among women without a previous caesarean, fetal distress was the most common reason for intrapartum caesareans in England, while failure to progress in labour was the most common reason for intrapartum caesareans in Queensland. For women with a previous caesarean, failure to progress was the most common reason for intrapartum caesarean in both countries.

While some similarities were identified between countries as to the most common reasons for caesarean, a number of key differences were also observed. Perhaps most notable were the apparent differences in the salience of previous caesarean section as the single main reason for caesarean. Compared to women in England, women in Queensland were more likely to have a pre-labour caesarean and to have an intrapartum caesarean due only to having previously had a caesarean. Although the proportion of women with a previous caesarean was higher in the Queensland sample, when those women were isolated, the proportion having a repeat caesarean due only to having previously had a caesarean remained higher in the Queensland sample.

While such vast differences could be the result of incongruent clinical standards, guidelines in Queensland and the UK [21,26] are aligned in their recommendations for women with a previous caesarean section. Both currently recommend discussion of the risks and benefits of different modes of birth, consideration of the capabilities of the facility, and responsivity to maternal preferences for mode of birth. However, there is a longer history of guidelines legitimising the role of maternal preferences in the UK than in Australia [38]. Such guidelines were first released in Queensland at approximately the same time as women in our sample gave birth, and represented a significant departure from existing documents that recommended such mode of birth decisions be guided by clinical expertise [39]. The recency of the shift towards responding to women’s preferences in Queensland may explain discrepancies in the practice patterns reported by women with a previous caesarean.

Discrepancies were also identified between countries in the reasons for caesarean among women without a previous caesarean. Intrapartum caesareans due to a failure to progress in labour were more likely among women in Queensland than women in England. While the term ‘failure to progress’ can incorporate a wide range of circumstances, these are typically inter-related and often characterised by a prolonged labour. Criteria for defining prolonged labour are similar in Queensland and the UK [40,41], however the only reference to management of delayed progress in labour in any current Queensland guideline is available in the *Normal Birth* guidelines and advice is restricted to ‘consulting an obstetrician’ [41]. Previous studies have demonstrated that international variation in caesarean rates is largely attributable to rates of caesarean among nulliparous women with singleton, term, and cephalic pregnancies [42]. The authors propose that differences in obstetric practice for the management of labour (e.g., use of oxytocin to correct dystocia) may be responsible for such variation [42]. The absence of clear guidance for intrapartum management of potential risk factors in Queensland is likely to result in variable practice that may not be based on current evidence. Women in Queensland were also at greater odds than women in England of having had a pre-labour caesarean due to suspected disproportion. Guidelines from the UK suggest that suspected disproportion alone should not be an indication for caesarean due to the limited reliability of methods to estimate infant size while in utero [21,38]. In Queensland and Australia there is not a single guideline for caesarean section, but rather, a collection of guidelines for specific populations or indications [26-29]. Currently there is not a clinical guideline for suspected cephalopelvic disproportion or suspected macrosomia, so it is unclear how decisions about mode of birth are made when such concerns are raised.

Although differing among primary pre-labour caesareans, maternal preference caesarean was reported at a low frequency by women in both countries (less than 1% of births). In both the UK and Australia, care providers are supported by professional bodies to perform a caesarean for maternal request in the absence of a medical indication if they consider the woman’s preference to be fully informed, and are comfortable performing the procedure [21,28]. Despite professional endorsement under given circumstances, maternal request as a sole reason does not account for the observed differences across countries in rates of caesarean section. Our findings are consistent with previous studies [19,43,44] that maternal request contributes to only a small proportion of the overall rates of caesarean section.

Carer or hospital recommendation was not provided in the list of reasons for caesarean for women in England; nor did any women spontaneously report this as the main reason for their caesarean. In Queensland, 1.5% of intrapartum caesareans and 6.5% of pre-labour onset caesareans were reported by women as being due to carer/hospital recommendation alone (i.e., without specifying concurrent clinical indications). Given the variation in measurement across the two countries, it remains difficult to determine whether the discrepant rates are indicative of true differences in practice between the countries.

Another possible explanation for the observed cross-cultural differences in practice patterns is differences in the training received by those supporting women through labour and birth. In England, more than half of women (56.3%) are cared for primarily by a midwife during labour and birth [24]. While a similar rate of midwife care (57.5%) is evident for the approximately 70% of Queensland women who birth in the public sector, a large majority of the 30% of Queensland women who birth in the private sector (89.6%) are cared for by an obstetrician [45]. In Queensland, women who birth in the private sector are more likely than women in the public sector to have a caesarean section, and these differences are not attributable to maternal risk or preference [46]. Further examination of the impact of training of the primary accoucheur, on both care decisions made during pregnancy or labour and the associated outcomes, is an important avenue for study in this field.

Strengths and limitations of the current study must be acknowledged. Unlike many previous studies that have used routinely collected data (such as hospital records or birth registrations) to examine rates of caesarean section [14,18,23], our findings are based on data reported by women and thus hinge on the reliability of this information. Despite this difference, the reported rates of caesarean section and assisted vaginal delivery in our samples vary little from national population data in both countries [24,25]. This is consistent with previous literature highlighting the congruence of maternal self-report and medical records on indicators such as mode of birth, reason for caesarean, reproductive and obstetric history, onset of labour, use of analgesia, perineal status after birth, and infant birthweight [30-33,47]. As with any method, self-report is not without bias and relies on women having been adequately informed regarding their treatment and being able to reliably recall this information. While only one study appears to have examined informant concordance of reason for caesarean, mismatch was mainly demonstrated for classifications reported by the clinician as ‘failed induction’, with women being more likely to provide the reason for the attempted induction as the indication for caesarean [33]. Thus discordance of indications for caesarean may be more likely when multiple factors are present, however reports are still well-aligned in such cases. Given that the focus of this paper was on examining differences between countries in women's self-reported indication, and any self-reported error is likely to be similar in both samples, this is unlikely to have influenced our main findings. It is possible that there are systematic differences between the countries in the classification and communication of reasons for caesarean, however the potential influence of this on how women ascribe reasons for their caesarean, along with the observed differences in maternal education, is unknown.

The response rates of the respective surveys may limit the generalisability of the findings. Other self-report population-level surveys of maternity care experience have achieved higher rates of response [48,49], however recruitment strategies relied on hospital and care provider involvement which may interfere with the perceived independence of the survey. While the women who participated in this study were not representative of the population of birthing women in their respective countries on some demographic measures, the respondent samples were largely representative on key clinical indicators examined in this study (e.g., mode of birth and experience of labour). Overrepresented characteristics in the sample that are often associated with increased likelihood of caesarean, such as maternal age in England and multiple pregnancy or private facility in Queensland, do not appear to have increased the observed rates for mode of birth. It is unclear how this may have affected the reporting of reasons for caesarean.

The measure of single (main) reason for caesarean, derived based on hierarchical ordering of clinical indications [see Additional file 1], may not have accounted for the possible complex interactions between indications. As already discussed, previous literature relating to indications for caesarean has relied on routinely collected data wherein a single indication for caesarean is provided by the attending clinician. How decisions are made when multiple indications are present or the consistency of approach between different clinicians is unknown. Most women in our sample provided only one or two reasons for their caesarean and where multiple reasons were provided this was often the pairing of a clinical indicator with maternal preference or carer/hospital recommendation. Decisions about coding were held consistent across the two countries to avoid artificial inflation of differences in main reason for caesarean. However, it should also be noted that the checklists provided to women to assess reasons for caesarean differed slightly between countries (see Table 1). While we have been intentionally cautious around interpretation of findings relating to carer/hospital recommendation and multiple pregnancy as reasons for caesarean, it is possible that the absence of these options may have altered how women responded to the question.

## Conclusions

Consistent with population statistics [24,25], the overall rate of caesarean section was higher in Queensland than in England in our samples, with differences particularly notable for pre-labour and repeat caesarean sections. Differences in the rate of primary caesarean section may be being driven by reasons such as failure to progress in labour and suspected disproportion. For women who had previously given birth by caesarean, those in Queensland were more likely to have had a repeat caesarean for this reason only, and very few attempted a vaginal birth. This is indicative of a cyclical effect whereby the higher rates of primary caesarean and lower rates of attempted VBAC lead to compounded increases in the rates of caesarean. In Queensland, the absence of a single, comprehensive clinical practice guideline for caesarean section may have resulted in more variation in care that is not based on current evidence and dominated by risk mitigation. Evaluation of practice in Queensland to determine the effects the recent implementation of specific clinical guidelines (e.g., VBAC) on care and rates of intervention, including caesarean birth, is an important avenue of further research.

## Abbreviations

VBAC: Vaginal birth after caesarean; UK: United Kingdom; CS: Caesarean section; GDM: Gestational diabetes.

## Competing interests

The authors declare that they have no competing interests.

## Authors’ contributions

All authors contributed to the study design and were involved in data collection. MR and SP coded the ‘other’ reasons for caesarean and all authors contributed to the development of the hierarchical algorithm for coding the single main reason for caesarean. SP and YM developed the analysis plan and interpreted the findings. RT provided critical direction for the framing of the Background and Discussion sections. SP conducted the analyses and drafted the manuscript. All authors provided critical review throughout the drafting process, approved a final version of the manuscript for submission, and are accountable for the accuracy and integrity of the findings.

## Pre-publication history

The pre-publication history for this paper can be accessed here:

http://www.biomedcentral.com/1471-2393/14/149/prepub

## Supplementary Material

Additional file 1 Hierarchical Algorithm for Coding Single (Main) Reason for Caesarean. Click here for file
