# Supplementary material for: Why 'down under' is a cut above: a comparison of rates of and reasons for caesarean section in England and Australia
Source: BMC Pregnancy Childbirth. 2014 Apr 26;14:149. doi: 10.1186/1471-2393-14-149 (PMC4021562; doi:10.1186/1471-2393-14-149)
Supplement: Additional file 1 — Hierarchical Algorithm for Coding Single (Main) Reason for Caesarean. [file 1471-2393-14-149-S1.docx]

**Additional Files**

**Additional File 1 - Hierarchical Algorithm for Coding Single (Main) Reason for Caesarean**

| **Self-reported Reason(s) for Caesarean** | **Coded Reason** |
| --- | --- |
| 1. Single reason only | Reason reported |
| 2. Single clinical reason (e.g., ‘fetal distress’) + ‘maternal preference’ | Clinical reason reported |
| 3. Single clinical reason (e.g., ‘fetal distress’) + ‘recommendation’ | Clinical reason reported |
| 4. ‘Breech presentation’ + any other reason(s) | ‘Breech presentation’ |
| 5. ‘Fetal distress’ + any other reason(s) | ‘Fetal distress’ |
| 6. ‘Failure to progress’ or ‘malpresentation’ or ‘disproportion’ (where caesarean was performed after labour onset) | ‘Failure to progress, malpresentation, obstruction/disproportion' |
| 7. ‘Failure to progress’ + other clinical reason (where caesarean was performed prior to labour onset) | Clinical reason reported |
| 8. ‘Malpresentation’ + other clinical reason (where caesarean was performed prior to labour onset) | Clinical reason reported |
| 9. ‘Disproportion’ + other clinical reason (where caesarean was performed prior to labour onset) | Clinical reason reported |
| 10. ‘Failure to progress’ only (where caesarean was performed prior to onset of labour) ^ | ‘Other’ |
| 11. ‘Malpresentation’ only (where caesarean was performed prior to onset of labour) | ‘Malpresentation’ |
| 12. ‘Disproportion’ only (where caesarean was performed prior to onset of labour) | ‘Suspected disproportion’ |
| 13. ‘Maternal health’ + ‘Fetal health’ | ‘Fetal health’ |
| 14. ‘Maternal health’ + ‘Fetal health’ + other reason(s) | ‘Fetal health’ |

Note. ^ A small number of women who did not experience a labour indicated the reason for their caesarean was ‘failure to progress in labour’. Due to ambiguity about the intended meaning, these cases were coded as ‘other’.
